# Cough-aerosol cultures of *Mycobacterium tuberculosis* in the prediction of outcomes after exposure. A household contact study in Brazil

**DOI:** 10.1371/journal.pone.0206384

**Published:** 2018-10-29

**Authors:** Carlos Acuña-Villaorduña, Luiz Guilherme Schmidt-Castellani, Patricia Marques-Rodrigues, Laura F. White, David Jamil Hadad, Mary Gaeddert, Jerrold J. Ellner, Kevin P. Fennelly, Moises Palaci, Reynaldo Dietze, Edward C. Jones-López

**Affiliations:** 1 Section of Infectious Diseases, Department of Medicine, Boston University School of Medicine and Boston Medical Center, Boston, Massachusetts, United States of America; 2 Mycobacteriology Laboratory, Núcleo de Doenças Infecciosas, Universidade Federal do Espírito Santo, Vitória, Brazil; 3 Núcleo de Doenças Infecciosas, Universidade Federal do Espírito Santo, Vitória, Brazil; 4 Department of Biostatistics, Boston University School of Public Health, Boston, Massachusetts, United States of America; 5 Pulmonary Clinical Medicine Section, Cardiovascular and Pulmonary Branch, Division of Intramural Research, National Heart, Lung, and Blood Institute, National Institutes of Health, Bethesda, Maryland, United States of America; 6 Global Health & Tropical Medicine - Instituto de Higiene e Medicina Tropical - Universidade Nova de Lisboa, Lisbon, Portugal; Public Health England, UNITED KINGDOM

## Abstract

**Background:**

*Mycobacterium tuberculosis* cultures of cough-generated aerosols from patients with pulmonary tuberculosis (TB) are a quantitative method to measure infectiousness and to predict secondary outcomes in exposed contacts. However, their reproducibility has not been established.

**Objective:**

To evaluate the predictive value of colony-forming units (CFU) of *M*. *tuberculosis* in cough aerosols on secondary infection and disease in household contacts in Brazil.

**Methods:**

Adult sputum smear+ and culture+ pulmonary TB cases underwent a standard evaluation and were categorized according to aerosol CFU. We evaluated household contacts for infection at baseline and at 8 weeks with TST and IGRA, and secondary disease.

**Results:**

We enrolled 48 index TB cases; 40% had negative aerosols, 27% low aerosols (<10 CFU) and 33% high aerosols (≥10 CFU). Of their 230 contacts, the proportion with a TST ≥10 mm at 8 weeks was 59%, 65% and 75%, respectively (p = 0.34). Contacts of high aerosol cases had greater IGRA readouts (median 4.6 IU/mL, IQR 0.02–10) when compared to those with low (0.8, 0.2–10) or no aerosol (0.1, 0–3.7; p = 0.08). IGRA readouts in TST converters of high aerosol cases (median 20 IU/mL, IQR 10–24) were larger than those from aerosol-negative (0.13, 0.04–3; p = o.o2). 8/9 (89%) culture+ secondary TB cases occurred in contacts of aerosol+ cases.

**Conclusion:**

Aerosol CFU predicts quantitatively IGRA readouts among household contacts of smear positive TB cases. Our results strengthen the argument of using cough aerosols to guide targeted preventive treatment strategies, a necessary component of current TB elimination projections.

## Introduction

Successful transmission of *Mycobacterium tuberculosis* results from a complex web of interactions between the source case, the exposed contact and the infecting pathogen within a variety of environments [1,2]. Together, these factors determine the number and viability of *M*. *tuberculosis* bacilli contained in cough-generated aerosols, the infectious moiety in tuberculosis (TB) [3,4]. Yet, despite long standing evidence for the latter, most of the evidence on TB transmission outcomes is based on the visualization of acid-fast bacilli (AFB) in sputum, which is still regarded as the definitive marker for infectiousness. However, in addition to poorly predicting transmission, sputa specimens fail to consider the complexities and stresses required for *M*. *tuberculosis* aerosolization, a necessary first step for successful transmission [1,5]

Over the last decade, our group has shown that the number of colony forming units (CFU) of *M*. *tuberculosis* cultured in cough-generated aerosols is a quantitative and more precise method for measuring source infectiousness and risk of infection in exposed contacts than sputum AFB smear microscopy. We have observed wide variability in aerosol CFU even among sputum AFB+ and culture-positive TB patients, [6] and found both qualitative and quantitative differences in tuberculin skin test (TST) and interferon gamma release assay (IGRA) readouts between contacts of aerosol-positive and aerosol-negative TB cases [7]. Significantly, in a follow-up study, household contacts of high aerosol (≥10 CFU) TB patients were at increased risk of incident TB disease [8]. Taken together, these data suggest aerosol CFU represent a promising marker of the infecting inoculum (e.g. inhaled dose), and that the inoculum size is a critical determinant modulating TB outcomes in humans, as observed in experimental TB models [5,9].

Whereas the aerosol collection method we use is validated [10, 11], the generalizability of our findings in exposed contacts is unknown. In our initial studies, the predictive value of aerosols was most evident in household contacts undergoing TST conversion, but less so in those that were TST-positive at baseline [6–7]. The utility of aerosols in settings with a lower TB prevalence, different environmental conditions (i.e. ambient humidity and temperature), and different *M*. *tuberculosis* strains is unknown. We conducted this household contact study to determine the reproducibility of cough aerosols to predict exposure outcomes in contacts of pulmonary TB patients.

## Methods

### Study population

This study was conducted at the Núcleo de Doenças Infecciosas (NDI) located in Vitória, the capital city of the State of Espírito Santo, Brazil. The NDI has organized a network of five laboratories in the metropolitan region of Vitória that serve a network of 16 TB clinics. The prevalence of HIV in the general population is <1%, and 7% in TB cases. The annual TB incidence in Espírito Santo is 38/100,000 inhabitants [12].

### Measurements

#### TB cases

Pulmonary TB patients identified through the NDI clinic network were eligible to participate, provided they fulfilled the following: 1) age ≥18 years with cough ≥3 weeks and 2) new TB episode with ≥1 sputum specimen with AFB ≥2+ with subsequent *M*. *tuberculosis* growth in culture. We excluded index cases who were HIV-infected, had a history of TB treatment, or who were too ill to consent, unable to understand, or to comply with the study protocol. To minimize differences in exposure time between study households, participating families were screened and enrolled within the first 2 weeks after the index case first presented to the municipal TB clinic. We collected clinical information and measured cough severity using a self-reported visual analog cough scale (VACS) [13], and the Leicester Cough Questionnaire (LCQ) [14], as reported [15]. We obtained three sputa specimens for AFB smear (auramine O fluorescent stain) [16], solid media (Ogawa-Kudoh) [17], and liquid (MGIT 960) cultures. The radiological extent of disease was graded on a four-category ordinal scale (normal, minimal, moderate and far-advanced) by an experienced radiologist [18]. We cultured *M*. *tuberculosis* from cough aerosols using the cough aerosol sampling system (CASS), as described [19]. Briefly, patients were instructed to cough into the CASS apparatus for two 5-minute periods separated by a 5-minute rest. CASS agar plates were read weekly for up to 6 weeks to count CFU of *M*. *tuberculosis*, the primary CASS outcome. All patients were offered TB treatment according to Brazilian guidelines [20].

#### Household contacts

We followed recommendations of the Brazilian National TB Program for household contact investigations [20]. Study staff visited dwellings to verify the identity of contacts, record clinical information, measure individual contact time with the index case, and to perform an environmental evaluation (crowding and ventilation). We recorded demographic and clinical characteristics of contacts and evaluated them for *M*. *tuberculosis* infection with both TST and IGRA, according to a predefined testing algorithm (S1 File) [15].

#### Secondary TB cases

In November 2017, we searched the Vitória TB Control Program records and the Brazilian National Notifiable Disease Information System (SINAN) database for the name, address, and date of birth of household contacts enrolled in the study to ascertain those that had developed subsequent TB; for those identified as TB cases, we registered date of diagnosis, AFB smear and culture results. We defined microbiologically confirmed TB as secondary TB cases among contacts with a positive culture for *M*. *tuberculosis*.

### Statistical methods

Our primary exposure variable was the cough aerosol status of the index TB patient living in the household. We categorized households into: aerosol negative (0 CFU), low aerosol (1–9 CFU), and high aerosol (≥10 CFU). We chose a 10 CFU cut-off because at this point we noted an increase risk in TST conversion in previous studies [6]. Our primary outcome was TST ≥10mm in contacts, using the maximum TST for each individual (e.g. 1^st^ or 2^nd^ TST), and completed an age-stratified analysis of TST results. We also performed a quantitative analysis of IGRA readouts using standard box plots to represent normalized IGRA values for each contact at 8–12 weeks. Our secondary outcome was microbiologically confirmed TB disease in contacts; given the small number of secondary TB cases, we categorized the exposure for this analysis as aerosol negative vs. aerosol positive. We calculated descriptive statistics to identify clinical and demographic differences between contact groups. To account for clustering within households, we calculated P values using generalized estimating equations or non-parametric tests if appropriate (SAS 9.1, SAS Institute, Inc., Cary, NC). Dataset is available in S1 Dataset.

### Ethical approvals

The study was approved by the Comitê de Ética em Pesquisa do Centro de Ciências da Saúde—Universidade Federal do Espírito Santo and the Comissão Nacional de Ética em Pesquisa (CONEP), and the Institutional Review Boards of Boston University Medical Center and New Jersey Medical School—Rutgers University (formerly UMDNJ). We obtained written informed consent and assent in Portuguese in accordance with age-specific ethical guidelines.

## Results

From April 2013 to June 2015, we screened 53 TB cases, but excluded 5 (10%) of them (Figure A in S1 File). Also, of the 253 eligible household contacts, 23 (9%) were excluded because of missing TST results. Therefore, the study population comprised 48 index TB cases and their 230 household contacts. Excluded contacts were similar to those included in terms of age (p = 0.35), gender (p = 0.57) and BCG vaccination scar (p = 0.78).

### Index TB cases and *M*. *tuberculosis* in cough aerosols

Index TB cases were mostly male (75%), with a median [interquartile range, IQR] age of 31 years [22–44], had a median duration of cough of 12 [7–24] weeks, 75% were sputum AFB 3+, and 69% had advanced disease on chest radiograph Table 1 and Table A in S1 File. Nineteen (40%) were aerosol-negative, 13 (27%) had low aerosols (1–9 CFU) and 16 (33%) had high aerosols (≥10 CFU); there was significant variability in CFU among aerosol-positive patients (median 31, range 1–333). Unlike sputum AFB, aerosol CFU was not associated with markers of pulmonary disease severity or bacterial load in sputum (Table 1).

**Table 1:** Characteristics of 48 index tuberculosis cases and their 230 household contacts according to the number of colony-forming units of *M*. *tuberculosis* in cough-generated aerosols and sputum acid-fast bacilli smear microscopy results.

Characteristic	Total	Aerosol negative (CFU = 0)	Aerosol positive		P^1^	Sputum AFB smear		P^1^
			Low aerosol (<10 CFU)	High aerosol (≥10 CFU)		1+ or 2+	3+	
Index Case Factors								
N	48	19 (40)	13 (27)	16 (33)		12 (25)	36 (75)	
Age	31 [22–44]	35 [22–50]	31 [22–44]	30 [22–43]	0.71	29 [23–38]	35 [22–45]	0.32
Gender								
Male	36 (75)	14 (74)	10 (77)	12 (75)	0.98	10 (83)	26 (72)	0.44
Female	12 (25)	5 (26)	3 (23)	4 (25)		2 (17)	10 (28)	
Karnofsky score								
80	24 (50)	9 (47)	4 (31)	11 (69)	0.12	6 (50)	18 (50)	1.0
90–100	24 (50)	10 (53)	9 (69)	5 (31)		6 (50)	18 (50)	
VACS	7 [5–9]	7 [5–9]	7 [5–9]	7 [5–9]	0.72	5 [4–6]	7 [5–8]	0.09
Cough peak flow^2^ (mL/min)	270 [150–370]	275 [170–370]	280 [200–400]	245 [120–335]	0.83	290 [230–420]	245 [120–360]	0.20
Chest radiograph					0.74			0.09
Normal/Minimal	3 (6)	2 (11)	1 (8)	0		2 (17)	1 (3)	
Moderate	12 (25)	4 (21)	3 (23)	5 (31)		1 (8)	11 (30)	
Advanced	33 (69)	13 (68)	9 (69)	11 (69)		9 (75)	24 (67)	
Cavitations					0.96			0.24
Absent	7 (15)	3 (16)	2 (15)	2 (13)		3 (25)	4 (11)	
present	41 (85)	16 (84)	11 (85)	14 (87)		9 (75)	32 (89)	
Sputum volume (mL)	5 [2–10]	3 [1–5]	2 [1–4]	7 [4–10]	0.09	5 [2–5]	5 [2–10]	0.85
Sputum AFB smear					0.78			
1+	3 (6)	2 (11)	0	1 (6)		-	-	
2+	9 (19)	4 (21)	2 (15)	3 (19)		-	-	
3+	36 (75)	13 (68)	11 (85)	12 (75)		-	-	
Sputum MGIT culture (DTP)	5 [4–7]	6 [4–9]	5 [4–7]	4 [4–6]	0.30	8 [6–27]	5 [4–6]	0.03
Aerosol CFU								
Median [IQR]	2 [0–45]	0	3 [2–5]	72 [45–102]	-	1 [0–29]	3 [0–58]	0.50
Mean {sd}	33.1 {65.2}	0	3.3 {2.2}	97 {82.7}	-	38.8 {94.7}	31.3 {56.6}	0.73
Range	[0–333]	[0–0]	[1–8]	[17–333]	-	[0–333]	[0–203]	
Household Contact Factors								
N	230	82	64	84		43	187	
Age	23 [15–42]	27 [15–48]	23 [15–44]	21 [13–40]	0.06	23 [16–44]	23 [13–41]	0.27
Gender					0.98			0.41
Male	95 (42)	32 (42)	27 (42)	36 (43)		16 (37)	79 (43)	
Female	130 (58)	45 (58)	37 (58)	48 (57)		27 (63)	103 (57)	
BCG scar N	125	51	45	29	0.31	16	109	0.75
Yes	105 (84)	41 (80)	37 (82)	27 (93)		13 (81)	92 (84)	
No/Uncertain	20 (16)	10 (20)	8 (18)	2 (7)		3 (19)	17 (16)	

Definition of abbreviations: AFB (acid-fast bacilli), CFU (colony forming units of *M*. *tuberculosis*), DTP (Days-to-positive in MGIT 960 culture), VACS (visual analog cough scale)

Values are median [IQR] or n (%) unless otherwise specified

Missing values (N): MGIT (17), sputum volume (12), contact age (6),

^1^ P values are Kruskal Wallis and Chi Square for index factors. For contact factors, we used generalized estimating equations (GEE).

^2^ The highest of 3 peak flow measurements was used for this analysis

### Household contacts and baseline TST results

**Fig 1:**
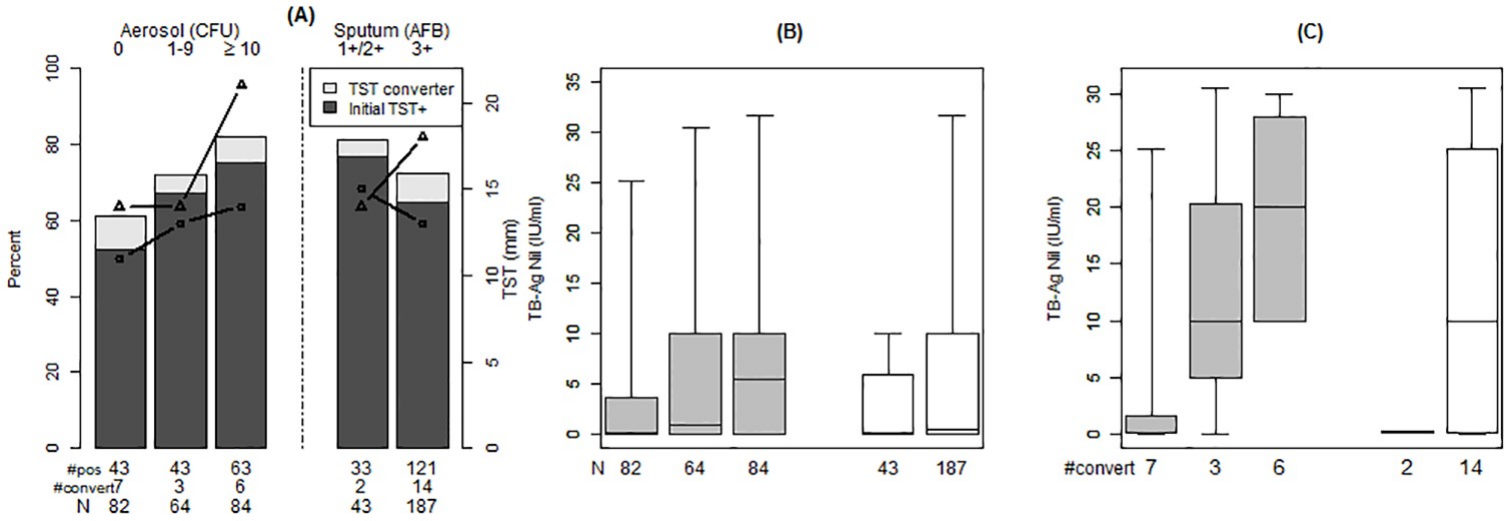
TST and IGRA results in 230 household contacts of TB patient according to the number of CFU of M. tuberculosis in cough-generated aerosols or AFB smear microscopy in the index TB case. **(A)** Histograms represent the proportion of contacts with a TST ≥10mm at baseline (black) and those with TST conversion (grey). The solid line represents the median TST diameter (mm) for contacts with TST ≥10mm at baseline (circles) and maximum TST for those with TST conversion (triangles) for each exposure group. **(B)** Standard box plots of maximum IGRA readouts in contacts of smear + culture + TB cases. P = 0.08 for aerosol CFU, p = 0.26 for AFB sputum smear **(C)** Standard box plots of IGRA readouts in contacts with TST conversion. P = 0.02 for aerosol CFU, p = 0.33 for AFB sputum smear.

Household contacts were young (median age 23 [15–42]), majority female (58%) and 84% had a BCG scar (Table 1). The number of contacts exposed to aerosol-negative, low, and high aerosol cases was 82 (36%), 64 (28%) and 84 (37%), respectively (Figure A in S1 File). At baseline (Table 2 and Fig 1, Panel A), the frequency of TST ≥10mm in contacts increased following a dose-response pattern as aerosol CFU increased: aerosol negative (59%), low aerosol (67%), and high aerosol (75%), respectively (p = 0.46); these results were unchanged when using a TST cut-off of 5mm or 15 mm. Similarly, in a quantitative analysis, the median TST diameter increased in parallel to aerosol CFU. In contrast, when using sputum AFB to risk-stratify the exposure, neither the proportion of contacts with TST ≥10 mm nor the TST diameter followed a dose-response pattern.

**Table 2:** Tuberculin skin test (TST) and interferon gamma release assay (IGRA) in household contacts according to the number of colony-forming units of *M*. *tuberculosis* in cough-generated aerosols and sputum acid-fast bacilli smear microscopy results.

Characteristic	Total	Aerosol negative (CFU = 0)	Aerosol positive		P^1^	Sputum AFB smear		P^1^
			Low aerosol (<10 CFU)	High aerosol (≥10 CFU)		1+ or 2+	3+	
Household Contact Factors								
N	230	82	64	84		43	187	
TST 1 (mm)								
Median [IQR]	13 [0–18]	11 [0–16]	13 [0–20]	14 [9–18]	0.46	15 [10–20]	13 [0–17]	0.13
Mean {sd}	11.0 {8.1}	9.8 {7.7}	11.6 {8.5}	12.5 {7.7}		13.4 {7.4}	10.8 {8.0}	
Range	[0–28]	[0–23]	[0–25]	[0–28]		[0–23]	[0–28]	
< 5mm	66 (29)	28 (34)	20 (31)	18 (21)	0.43	8 (19)	58 (31)	0.29
≥5mm	164 (71)	54 (66)	44 (69)	66 (79)		35 (81)	129 (69)	
< 10mm	76 (33)	34 (41)	21 (33)	21 (25)	0.33	10 (23)	66 (35)	0.26
≥10mm	154 (67)	48 (59)	43 (67)	63 (75)		33 (77)	121 (65)	
<15mm	134 (58)	56 (68)	33 (52)	45 (54)	0.35	18 (42)	116 (62)	0.04
≥15mm	96 (42)	26 (32)	31 (48)	39 (46)		25 (58)	71 (38)	
TST max^2^ (mm)								
Median [IQR]	14 [10–18]	14 [4–16]	14 [0–20]	15 [12–20]	0.22	15 [12–20]	14 [8–18]	0.24
Mean {sd}	12.7 {7.6}	11.3 {7.2}	12.5 {8.3}	14.2 {7.1}		14.1 {6.7}	12.3 {7.7}	
Range	[0–28]	[0–23]	[0–25]	[0–28]		[0–23]	[0–28]	
< 5mm	49 (21)	21 (26)	17 (27)	11 (13)	0.21	5 (12)	44 (24)	0.21
≥5mm	181 (79)	61 (74)	47 (73)	73 (87)		38 (88)	143 (76)	
< 10mm	56 (24)	24 (29)	17 (27)	15 (18)	0.41	8 (19)	48 (26)	0.41
≥10mm	174 (76)	58 (71)	47 (73)	69 (82)		35 (81)	139 (74)	
<15mm	124 (54)	53 (65)	32 (50)	39 (46)	0.22	17 (40)	107 (57)	0.05
≥15mm	106 (46)	29 (35)	32 (50)	45 (54)		26 (60)	80 (43)	
IGRA max (IU/ml)								
N	116	34	22	60		27	89	
Median [IQR]	0.3 [0–10]	0.09 [0–3.7]	0.9 [0.01–10]	5.5 [0.01–10]	0.08	0.01 [0–5.8]	0.4 [0.01–10]	0.26
Mean {sd}	4.6 {7.2}	2.9 {5.2}	4.8 {6.2}	8.4 {10.3}		2.7 {4.2}	5.1 {7.5}	
Range	[0–32]	[0–25.1]	[0–31]	[0–32]		[0–10]	[0–32]	
< 0.35	58 (51)	29 (60)	20 (48)	9 (38)	0.50	10 (63)	48 (49)	0.51
≥0.35	56 (49)	19 (40)	22 (52)	15 (62)		6 (37)	50 (51)	
<10	81 (71)	41 (85)	27 (64)	13 (54)	0.12	13 (81)	68 (69)	0.43
≥10	33 (29)	7 (15)	15 (36)	11 (46)		3 (19)	30 (31)	
IGRA delta								
N	58	32	12	14		7	51	
Median [IQR]	0.01 [0–0.04]	0 [0–0.02]	0.02 [0.01–0.1]	0.03 [0.01–20.1]	0.09	-0.03 [-.01–0.1]	0.01 [-0.03–0.04]	0.14
Mean {sd}	2.3 {8.3}	-0.6 {2.7}	2.7 {8.8}	8.5 {12.4}		-0.9 {2.6}	2.7 {8.7}	
Range	[-8.4–30.5]	[-8.3–7.2]	[-0.03–30.5]	[-2.8–28.9]		[-6.9–0.7}	[-8.3–30.5]	

Definition of abbreviations: IGRA (interferon gamma release assay, Quantiferon Gold In-Tube), TST (tuberculin skin testing).

Values are median [IQR] or n (%) unless otherwise specified

Missing values (N): IGRA (114), IGRA delta (172).

^1^ P values are estimated using generalized estimating equations (GEE).

^2^ The maximum TST value (TST1 or TST2) was used for each individual

### TST conversion in contacts

Seventy-six (33%) contacts had TST <10mm at baseline and were therefore at risk of TST conversion (Fig 1, Panel A). Of these, 34/82 (41%), 21/64 (33%) and 21/84 (25%) were contacts of aerosol-negative, low and high aerosol TB patients, respectively (p = 0.46). By 8–12 weeks after enrollment, 16 (21%) contacts had undergone TST conversion. Of these, 7 (21%) were contacts of aerosol-negative, 3 (14%) of low and 6 (29%) of high aerosol cases (p = 0.52). The median diameter in TST converters of high aerosol (21 mm [IQR 20–24]) patients was larger than in those of aerosol-negative (14 mm [IQR 14–16], P = 0.02) patients.

### IGRA readouts in household contacts

To examine the possibility of TST confounding by BCG and boosting, we analyzed infection outcomes according to IGRA. Qualitatively, the proportion of individuals with IGRA+ at study conclusion (8–12 weeks) followed a similar aerosol dependent dose-response pattern to that observed with TST results (Table 2). Similarly, quantitative IGRA readouts showed a strong dose-response pattern according to aerosol CFU, both when measured as mean maximum IGRA values per contact (Fig 1, Panel B; P = 0.08) or, in TST converters only (Fig 1, Panel C; P = 0.02). As observed with TST results, these quantitative IGRA differences were not apparent when using sputum AFB to risk-stratify the exposure (Table 2 and Fig 1).

### Age stratified analysis of TST results

**Fig 2:**
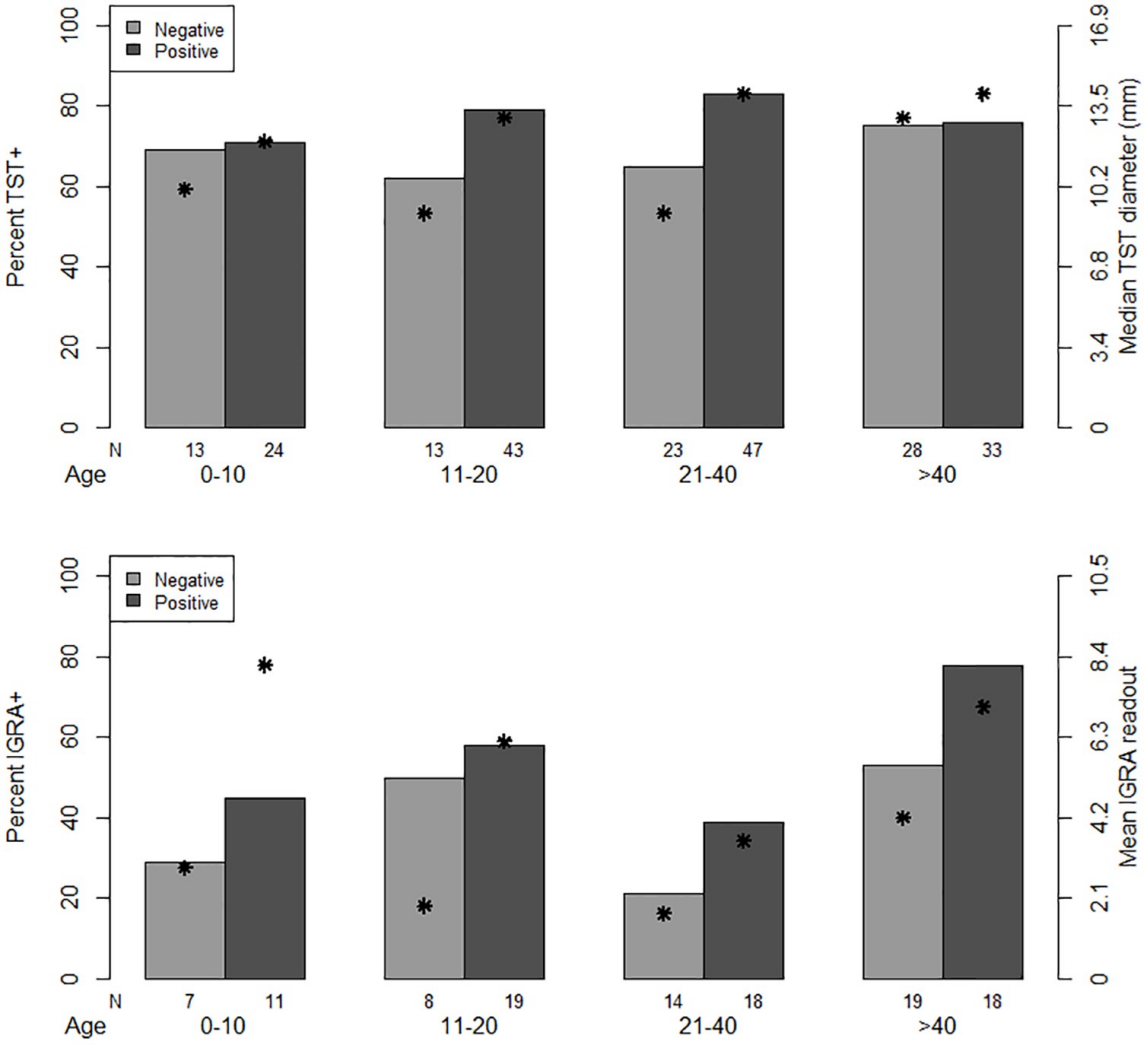
Proportion of household contacts with a positive (≥10mm) tTST and IGRA at study completion (8–12 weeks) by contact age group and CFU of *M*. *tuberculosis* in cough-generated aerosols in the index case: Negative (CFU = 0) or positive (CFU ≥1). The “N” under each histogram indicates the number of contacts within each age-exposure category. The stars (*) represent the median TST or mean IGRA readout associated with each group.

To further elucidate the potential effect of BCG on TST and indirectly estimate community-based transmission, we performed an age-stratified analysis of TST results. The proportion of contacts with TST ≥ 10 mm was similar across all ages, 0–10 years 26/37 (70%), 11–20 years 42/56 (75%), 21–40 years 54/70 (77%) and > 40 years 46/61 (75%). However, except for young children (0–10 years) where BCG could be influencing TST results, the prevalence of infection across contact age groups was consistently lower in contacts of aerosol-negative patients when compared to contacts of aerosol-positive patients, suggesting TST-positive results in the former likely reflect cumulative community exposures. Further, TST induration sizes and IGRA readouts were consistently larger for aerosol-positive HHCs in each age group (Fig 2).

### Secondary TB disease in household contacts

After a median follow-up of 3.6 years, 13 of 230 (5.6%) contacts developed secondary TB disease, of which 9 (4%) were culture-positive cases. Of the latter, 1/82 (1.2%) was in contacts of aerosol-negative cases and 8/148 (5.4%) in contacts of aerosol-positive cases (p = 0.176). The corresponding incidence of culture-proven secondary TB disease was 356 cases per 100,000 (95% CI 50–2,331) and 1,439 (95% CI 719–2,878), respectively. When all 13 secondary TB cases were included (4/82 [5%] vs. 9/148 [6%]), the association between positive aerosols and risk of secondary TB disease was weaker (p = 0.73).

## Discussion

In this household contact study in a setting with moderate TB incidence, we found that contacts of high aerosol TB patients were more likely to become infected and had larger TST and IGRA readouts compared to contacts of low and negative aerosol TB cases. Moreover, albeit limited by a small sample size, most cases of culture-proven secondary TB in contacts clustered around aerosol–positive index TB cases. Data from this study are consistent with our initial study in Uganda where contacts of high aerosol cases were also more frequently infected, displayed larger TST and IGRA readouts [7], and were at increased risk of incident TB disease (8). Taken together, these findings confirm that aerosol CFU is a promising marker of transmission risk and strengthens our hypothesis that the inoculum size is important in modulating TB outcomes after exposure in humans [5].

Close contacts of pulmonary TB cases are known to have increased risk of TB infection and disease when compared to the general population [2]. However, there is abundant both experimental and epidemiological evidence of marked variability in risk, as most secondary infections and disease cases cluster around a minority of TB cases [2–4]. Our findings that only a minority of sputum AFB+ (and culture+) TB cases naturally produce viable aerosolized *M*. *tuberculosis* during cough- is consistent with the existence of disease superspreaders [21]. As such, measuring cough aerosols allows identification of a subset of individuals at a particularly high risk of infection and disease, presumably as a result of a larger infectious dose. It follows that these contacts would benefit most of preventive therapy, while also increasing the efficiency of household contact investigations by redefining the population at highest risk. As we observed previously, these findings were not apparent through the prism of two standard indicators of infectiousness such as sputum AFB and lung cavitation on chest radiograph suggesting that risk stratification is limited without aerosols, the infectious moiety in TB [3].

In this study, we observed a dose response pattern in prevalent TB infection per aerosol group, an association that was not observed in Uganda. This suggests the aerosol phenotype is stable over time, as the 1^st^ TST results reflect the index cases’ infectiousness prior to study initiation. Interestingly, the proportion of aerosol-negative contacts with TST ≥10 mm at baseline was 59%, which is above the expected level of community transmission in Brazil [22]–indicating TST positivity does occur after exposure to aerosol-negative TB disease. However, whereas there was variability in individual results, the median IGRA in contacts of aerosol-negative TB patients was close to zero, suggesting that a TST+/IGRA- result in these contacts may be measuring a qualitatively different infection when compared to a comparatively positive TST result that is also accompanied by a robust IGRA response in contacts of aerosol-positive patients.

Some differences from our initial Ugandan studies [6–8] are noteworthy. First, we found a higher rate of aerosol production (60% vs 36%) in TB cases from Brazil. We suspect this is because in this study most patients were sampled prior to initiating antituberculous treatment. The rapid decrease of culturable organisms in aerosols is consistent with both experimental and clinical studies showing a marked reduction in infectiousness promptly after chemotherapy is started, even when sputum AFB smear and culture remain positive [3, 23,24]. Second, we did not find a significant difference in TST conversion per aerosol groups, but rather observed the effect of aerosols on TST positivity at baseline. Given the small number of TST converters in this study (n = 16), our study was likely underpowered to detect differences in TST conversion. Nevertheless, as observed in Uganda, IGRA readouts among Brazilian TST converters of high aerosol cases were significantly higher than those of aerosol-negative cases–suggesting that some conversions in aerosol-negative contacts could be due to TST boosting or low dose infection, and hence, presumably carry a lesser risk of progression to TB disease. An alternative explanation is that a delayed IGRA response is due to a less intense infectious exposure [25]. Finally, Ugandan contacts had a higher frequency of TST ≥10 mm and larger TST induration sizes, likely reflecting increased community transmission and more advanced disease in TB cases in the former setting.

We used TST as our primary outcome because it is the most widely used test to diagnose *M*. *tuberculosis* infection in resource constrained settings and to be consistent with our prior Ugandan study. Although BCG is known to produce false positive TST results, the effect is minimal after 10 years of age [26]. Further, 75% of HHCs had TST ≥ 10 mm compared with 53% of IGRA ≥ 0.35 UI/ml which is consistent with other household contact studies in developing countries whereas TST often yields more positive results compared with IGRA [27–28].

We have now accumulated enough evidence to demonstrate that our findings are neither spurious nor related to the aerosol collector device used [10]. To date, our group has collected aerosols in more than 25o patients from The United States, Uganda, and Brazil, and others have validated the CASS apparatus in South Africa [11]. Taken together, our data confirm the marked variability in TB transmission described in seminal studies [3] and in multiple epidemiological studies in both low and high burden settings [29,30]. Future studies should evaluate the use of exposure-based interventions where targeted preventive therapy is provided based on the ability of the TB index case to aerosolize *M*. *tuberculosis* rather than in the TST/IGRA status of the contacts and compare the efficacy and cost-effectiveness of such a strategy against universal preventive therapy, gene signatures blood biomarkers [31] and predictive scores [32–34].

Our study has limitations. In its current form, the CASS device is designed as a research tool rather than a point of care test, which limits its ability to be used at peripheral health care centers in developing countries where most TB cases are concentrated. Future designs should try to improve its applicability in resources constrained settings and to incorporate polymerase chain reaction (PCR) technologies to more rapidly identify aerosolized *M*. *tuberculosis*, although this improvement in time-to-result may not adequately reflect bacterial viability in aerosols [35]. Importantly, in the present study, the number of CFU in aerosols after two weeks of growth in cultures did not alter the final categorization (e.g. negative, low or high), suggesting that exposure-based interventions may be implementable in a timely manner. We had an important number of missing IGRA results in contacts; selection bias may have been introduced if differential IGRA levels were seen on contacts of high aerosol TB cases. We did not have access to genotypic testing of *M*. *tuberculosis* isolates to confirm that secondary TB cases originated from the index TB case in the household; however, Vitória is a setting with moderate TB incidence and thus we expect that most of the secondary TB cases originated from the household TB index case. Finally, our study was underpowered.

In conclusion, cultures of *M*. *tuberculosis* from cough-generated aerosols predict risk of infection, as measured by both qualitative and quantitative TST and IGRA readouts. Our results further strengthens the argument of using cough aerosols to implement targeted preventive therapy, a necessary component for current TB elimination targets.

## Supporting information

S1 File **Table A**: Additional Characteristics of 48 Index Tuberculosis Cases and their 230 Household Contacts **Figure A**: Study profile. IC = Index tuberculosis (TB) cases; HHC = Household contacts; TST = Tuberculin skin test; IGRA = Interferon gamma release assay (Quantiferon Gold In-Tube).
(DOCX)

S1 Dataset Database of 48 index tuberculosis cases and their 230 household contacts. (XLSX)
