# Supplementary material for: Cough-aerosol cultures of Mycobacterium tuberculosis in the prediction of outcomes after exposure. A household contact study in Brazil
Source: PLoS One. 2018 Oct 29;13(10):e0206384. doi: 10.1371/journal.pone.0206384 (PMC6205616; doi:10.1371/journal.pone.0206384)
Supplement: S1 File — Table A: Additional Characteristics of 48 Index Tuberculosis Cases and their 230 Household Contacts Figure A: Study profile. IC = Index tuberculosis (TB) cases; HHC = Household contacts; TST = Tuberculin skin test; IGRA = Interferon gamma release assay (Quantiferon Gold In-Tube). (DOCX) [file pone.0206384.s001.docx]

**Tables A**: Additional Characteristics of 48 Index Tuberculosis Cases and their 230 Household Contacts According to the Number of Colony-Forming Units of *M. tuberculosis* in Cough-Generated Aerosols and Sputum Acid-fast Bacilli Smear Microscopy Results in Vitória, Brazil.

| **Characteristic** | **Total** | **Aerosol negative**  **(CFU=0)** | **Aerosol positive** | | **P** | **Sputum AFB smear** | | **P** |
| --- | --- | --- | --- | --- | --- | --- | --- | --- |
|  |  |  | **Low aerosol**  **(<10 CFU)** | **High aerosol**  **(≥10 CFU)** |  | **1+ or 2+** | **3+** |  |
| Index Case Factors | |  |  |  |  |  |  |  |
| N | 48 (100) | 19 (40) | 13 (27) | 16 (33) |  | 12 (25) | 36 (75) |  |
| Body mass index (Kg/m^2^) | 20 [18-22] | 20.8 [17.8-23.3] | 18.7 [18-19.7] | 20.7 [18.4-22.4] | 0.49 | 20.1 [18-22.8] | 20 [18.1-21.9] | 0.96 |
| Weeks sick before enrolment | 12 [7-24] | 12 [10-32] | 12 [4-24] | 12 [7-18] | 0.76 | 12 [8-16] | 12 [6-24] | 0.87 |
| Number of contacts | 5 [4-6] | 4 [4-5] | 6 [4-6] | 5 [4-6] | 0.57 | 4 [3-5] | 5 [4-6] | 0.06 |
| Cough strength  Weak  Strong | 29 (60)  19 (40) | 11 (58)  8 (42) | 7 (54)  6 (46) | 11 (69)  5 (31) | 0.69 | 9 (75)  3 (25) | 20 (56)  16 (44) | 0.23 |
| LCQ | 14 [11-16] | 15 [11-15] | 14 [11-15] | 13 [11-16] | 0.88 | 15 [11-16] | 14 [11-15] | 0.31 |
| Days on TB treatment | 0 [0-0] | 0 [0-0] | 0 [0-0] | 0 [0-0] | 0.46 | 0 [0-0] | 0 [0-0] | 0.10 |

Definition of abbreviations: AFB (acid-fast bacilli), CFU (colony forming units of *M. tuberculosis*), LCQ (Leicester Cough Questionnaire)

Values are median [IQR] or n (%) unless otherwise specified

^1^ P values are estimated using Chi square and Fisher exact test (index factors)

^2^ Cough strength at the time of aerosol collection (subjective evaluation by technician).

**Figure A**

Eligible Families

N=53

IC exclusions (N=5):

- Less than 3 HHC (n=2)

- No CASS result (n=2)

- Information not available (n=1)

Eligible Cohort

IC: N=48

HHC: N=253

HHC exclusions (N=23):

- Refused initial TST (n=17)

- Refused second TST (n=6)

Study Cohort

IC: N=48

HHC: N=230

No aerosol

IC: N=19 / HHC: N=82

- 1st TST ≥10mm: 48 (59%)
- TST conversion: 7 (9%)
- IGRA+: 40%
- Median IGRA: 0.05 IU/mL

High aerosol

IC: N=16 / HHC: N=84

- 1^st^ TST ≥10mm: 63 (75%)
- TST conversion: 6 (7%)
- IGRA+: 62%
- Median IGRA: 5.5 IU/mL

Low aerosol

IC: N=13 / HHC: N=64

- 1^st^ TST ≥10mm: 43 (67%)
- TST conversion: 3 (5%)
- IGRA+: 52%
- Median IGRA: 0.9 IU/mL
